# Case Report: Coexistence of Alzheimer-Type Neuropathology in Fragile X-Associated Tremor Ataxia Syndrome

**DOI:** 10.3389/fnins.2021.720253

**Published:** 2021-09-17

**Authors:** Maria Jimena Salcedo-Arellano, Desiree Sanchez, Jun Yi Wang, Yingratana A. McLennan, Courtney Jessica Clark, Pablo Juarez, Andrea Schneider, Flora Tassone, Randi J. Hagerman, Verónica Martínez-Cerdeño

**Affiliations:** ^1^Department of Pediatrics, University of California, Davis, Sacramento, CA, United States; ^2^Department of Pathology and Laboratory Medicine, University of California, Davis, Sacramento, CA, United States; ^3^Medical Investigation of Neurodevelopmental Disorders (MIND) Institute, University of California, Davis, Sacramento, CA, United States; ^4^Institute for Pediatric Regenerative Medicine and Shriners Hospitals for Children Northern California, Sacramento, CA, United States; ^5^Center for Mind and Brain, University of California, Davis, Davis, CA, United States; ^6^Department of Biochemistry and Molecular Medicine, University of California, Davis, Sacramento, CA, United States

**Keywords:** FXTAS, Alzheimer-type dementia, *FMR1* gene, CGG expansion, *APOE* ε4 allele, neurodegeneration, cognitive decline

## Abstract

This case documents the co-occurrence of the fragile X-associated tremor ataxia syndrome (FXTAS) and Alzheimer-type neuropathology in a 71-year-old premutation carrier with 85 CGG repeats in the fragile X mental retardation 1 (*FMR1)* gene, in addition to an apolipoprotein E (*APOE)* ε4 allele. FXTAS and Alzheimer's Disease (AD) are late-onset neurodegenerative diseases that share overlapping cognitive deficits including processing speed, working memory and executive function. The prevalence of coexistent FXTAS-AD pathology remains unknown. The clinical picture in this case was marked with rapid cognitive decline between age 67 and 71 years in addition to remarkable MRI changes. Over the 16 months between the two clinical evaluations, the brain atrophied 4.12% while the lateral ventricles increased 26.4% and white matter hyperintensities (WMH) volume increased 15.6%. Other regions atrophied substantially faster than the whole brain included the thalamus (−6.28%), globus pallidus (−10.95%), hippocampus (−6.95%), and amygdala (−7.58%). A detailed postmortem assessment included an MRI with confluent WMH and evidence of cerebral microbleeds (CMB). The histopathological study demonstrated FXTAS inclusions in neurons and astrocytes, a widespread presence of phosphorylated tau protein and, amyloid β plaques in cortical areas and the hippocampus. CMBs were noticed in the precentral gyrus, middle temporal gyrus, visual cortex, and brainstem. There were high amounts of iron deposits in the globus pallidus and the putamen consistent with MRI findings. We hypothesize that coexistent FXTAS-AD neuropathology contributed to the steep decline in cognitive abilities.

## Introduction

Fragile X-associated tremor/ataxia syndrome (FXTAS) is a late-onset neurodegenerative disease characterized by neurological involvement including cerebellar ataxia, intention tremor, neuropathy, parkinsonism, executive dysfunction, and cognitive deficits. Clinical symptoms usually become apparent between the sixth and seventh decade of life. Carrying the premutation (55–200 CGG repeats) in the *FMR1* gene located in the X chromosome confers the genetic background for the development of FXTAS in ~40% of male and 16% of female carriers (Hagerman and Hagerman, 2016). Most of the affected patients face a slow but steady progression of motor and cognitive deficits. During the disease's final stages, ~15–25 years after the onset of symptoms, the patients are unable to perform basic activities of daily living and lose their independence. Cognitive decline leading to dementia is seen in ~50% of male patients with FXTAS (Seritan et al., 2016). Prior reports have introduced epigenetic/environmental risk factors related to an early presentation, greater severity of symptoms or faster progression of FXTAS including chronic use of addictive substances (Martinez-Cerdeno et al., 2015, Muzar et al., 2014, Muzar et al., 2015), anesthetics/general surgery (Ligsay et al., 2019), and exposure to environmental neurotoxins (Saldarriaga et al., 2019). Here we report an unusual case with a dramatic progression of cognitive decline associated with disinhibition in a patient with FXTAS and an *APOE* ε4 allele. The patient died within 10 years of clinical involvement. He was evaluated clinically, and he had postmortem imaging and tissue examination demonstrating co-occurrence of Alzheimer changes.

## Case Description

The patient initially presented to us at age 67 with a history of both tremor and balance difficulties. He was confirmed to have 85 CGG repeats and 4 daughters with the *FMR1* premutation as well as a niece and nephew with fragile X syndrome. Before developing FXTAS, he was noted to be athletic and an extremely successful professional.

After the loss of his father, at age 61, he developed bereavement-related depression, however, he did not receive antidepressants nor did he have therapy or counseling for his depression. Soon after, he began to present short-term memory problems, motor deficits including tremor and balance problems, as well as neuropathy in his lower extremities. As his hand tremor worsened, he also developed a head tremor. By age 62, his worsening short-term memory forced him to retire from his business. He began to have long lapses in his conversations as his word retrieval became worse. He had an episode of wandering at age 67 away from his hotel and he was disoriented, a subsequent evaluation with a CAT scan was normal. He also had frequent falls and at age 69 he fell but he did not have significant head trauma, he was discharged after neurological evaluation and observation in the emergency room.

His past medical history included episodes of vertigo which began at age 57. These episodes usually lasted a few minutes except for two episodes that were associated with muscle weakness and lasted a day. He also had a history of atrial fibrillation (Afib) diagnosed at age 57, requiring cardioversion at presentation. Recurrent Afib was treated with propafenone 150 mg bid and warfarin. Additional medications included pravastatin 20 mg a day, vitamins, and antioxidants. He also had 2 hospitalizations for severe pneumonia.

On his initial exam at age 67, his hearing was mildly decreased bilaterally, and his smile was asymmetrical with less tone on the left. His finger joints were swollen, and he had arthritic changes and nodules on the upper aspect of his fingers. His reflexes were hypoactive and absent at the ankles and vibration sense was diminished in the feet. He had prominent primitive reflexes including grasp, glabellar, snout and palmomental reflexes. He had an intention tremor bilaterally, a hand positional tremor bilaterally, and a mild “yes yes” head tremor. He had significant dysdiadochokinesis bilaterally and was unable to tandem walk with significant ataxia on heel to shin movements. Cognitive testing is shown in Table 1. Treatment with memantine and donepezil, and a follow-up with a geriatric psychiatrist and physical therapy was recommended for ataxia.

**Table 1:** Summary of neuropsychiatric results.

		**2016 Score**	**2016 Percentile rank**	**2017 Score**	**2017 Percentile rank**	**Description**
**WAIS-IV**						
	Verbal IQ	70	2	54	0.1	Borderline (2016), Extremely Low (2017)
	Performance IQ	65	1	63	1	Extremely Low
	Full Scale IQ	62	1	51	0.1	Extremely Low
	VCI	70	2	54	0.1	Extremely Low
	PRI	65	1	63	1	Extremely Low
	WMI	71	3	53	0.1	Extremely Low
	PSI	65	1	59	0.3	Extremely Low
WMS-IV^*^						
	Auditory Memory	–	–	58	0.3	Extremely Low
	Visual Memory	–	–	45	<0.1	Extremely Low
	Immediate Memory	–	–	54	0.1	Extremely Low
	Delayed Memory	–	–	54	0.1	Extremely Low
**BDS-2**						
		–	–	2	–	Severe difficulties with executive functioning
**MMSE**						
		–	–	15	–	Significant impairment in cognitive functioning

* *WMS Scores are provided as Scaled scores*.

*Based on the neuropsychological results and the caregiver's report on the patient's decline, a diagnosis of Major Neurocognitive Disorder Due to Another Medical Condition (F02.80, 294.10, DSM- 5) was probable. WAIS-IV, Wechsler Adult Intelligence Scale, 4th Edition; VCI, Verbal Comprehension Index; PCI, Perceptual Reasoning Index; WMI, Working Memory Index; PSI, Processing Speed Index; WMS-IV, Wechsler Memory Scale, 4th Edition; BDS-2, Behavior Dyscontrol Scale 2nd Edition; MMSE, Mini-Mental-State Exam*.

He was followed 2 years later, at age 69, with worsened tremors and swallowing problems. Although he was treated with memantine and donepezil his cognition had worsened. He had become more disinhibited in public and had aggressive behavior at night, so he was started on quetiapine to aid in sleep.

On exam, he had a coarse intention, positional and resting tremor in addition to his head tremor. He had masked facies, bradykinesia, and a cervical dystonia with his head tilted to the left. His reflexes were decreased and absent in the ankles and he continued to have signs of neuropathy with decreased pinprick and vibration sensations. He had marked primitive reflexes as previously noted. Cognitive testing (Table 1) demonstrated a major neurocognitive disorder and the results from the Structured Clinical Interview for DSM-IV (SCID) diagnosed Social Phobia (F40.1, 300.23). A trial with carbidopa/levodopa for emerging parkinsonian features was recommended. A comprehensive review of family history at this time revealed a maternal uncle who died from AD in his 50s; based on this new information genetic testing for *APOE* was carried out to evaluate the patient's predisposition for AD.

Although carbidopa/levodopa was initially beneficial, over the next 2 years he gradually developed worsening of his symptoms of tremor, weakness and falling until he was bedridden. He had episodes of staring which were thought to be transient ischemic attacks, but an electroencephalogram demonstrated right and left temporal spikes and a disorganized background rhythm. Due to swallowing problems, he was hospitalized with aspiration pneumonia at age 71. A percutaneous endoscopic gastrostomy (PEG) tube was placed for nutrition. An echocardiogram taken at the time of hospitalization showed mild left ventricular hypertrophy and tricuspid regurgitation as well as a small pericardial effusion. He was eventually transferred to hospice and needed continuous oxygen supply before he passed away at age 71.

## Diagnostic Assessment

### Neuropsychiatric Assessment

Cognitive testing is listed in Table 1 (WAIS-IV, Wechsler Adult Intelligence Scale, 4th Edition)(Wechsler, 2008). An assessment of memory skills (WMS-IV, Wechsler Memory Scale, 4th Edition)(Wechsler, 2009) and several other neuropsychological assessments were also conducted (Table 1) to evaluate executive function and general cognitive status [Behavior Dyscontrol Scale 2nd Edition(BDS-2) (Grigsby and Kaye, 1996), Mini-Mental-State Exam (MMSE)] (Folstein et al., 1975). His memory and executive function abilities demonstrated significant decline over the 2 clinical visits. The examiner noted that he often stared blankly during the second assessment, and it was not fully clear whether he understood the questions properly.

### Neuroimaging

Both *in vivo* and postmortem MRI were acquired on a Siemens Trio 3T MRI scanner (Siemens Medical Solutions, Erlangen, Germany) using a 32-channel headcoil. For postmortem MRI, the right hemisphere was immersed in an inert proton-free fluid, 3M fluorinert electronic liquid (FC-770, Parallax Technology, Inc.) during the acquisitions of T2 and multi-echo T2^*^ images.

**Figure 1:**
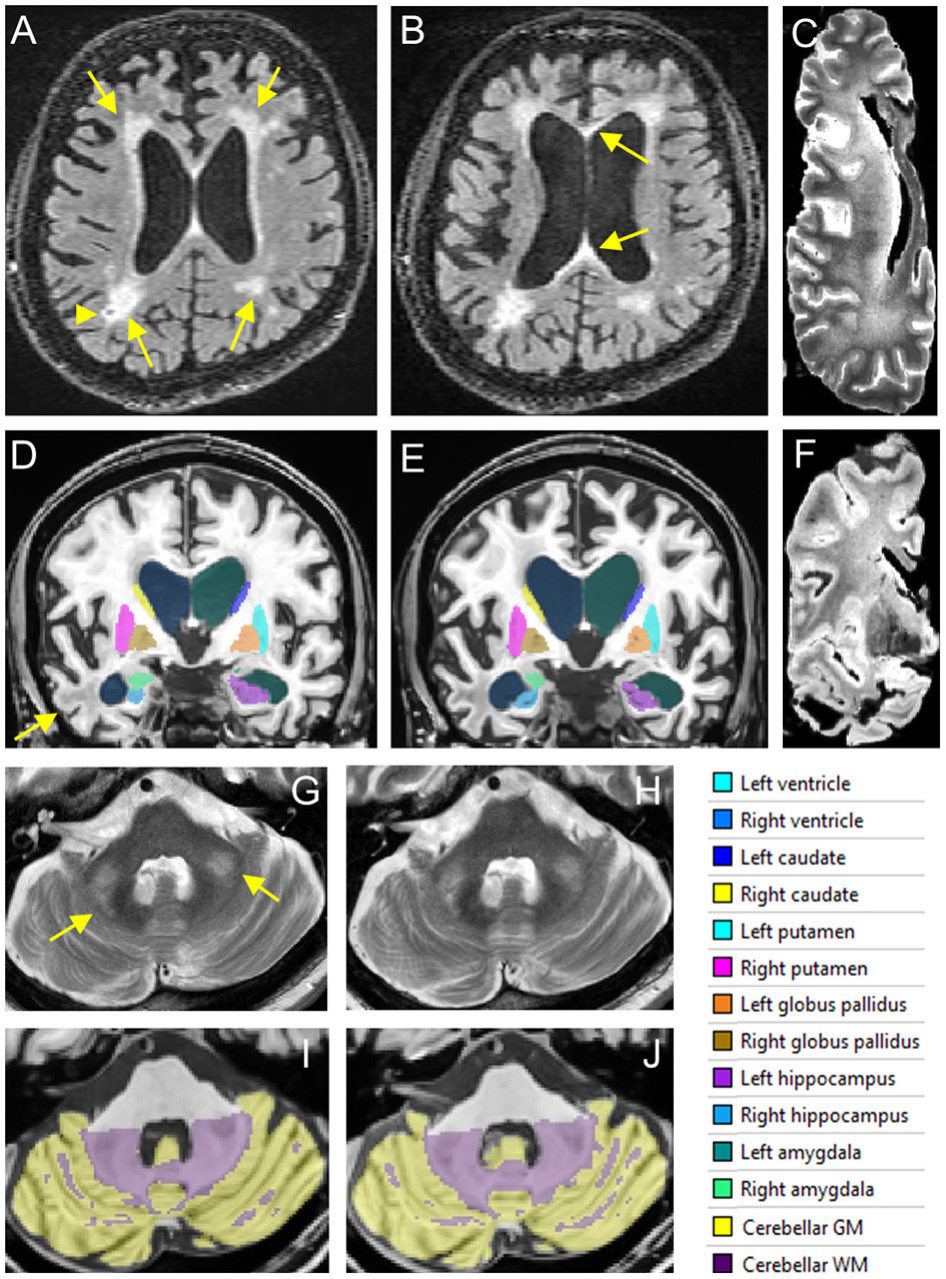
*In vivo* and postmortem MRI showing white matter lesions, brain atrophy, and enlarged ventricles. **(A**–**C)** FLAIR scans acquired in 2016 **(A)** and 2017 **(B)** show periventricular WMHs (arrows in **A**) and a lacune (arrowhead) in right parietal white matter. The corpus callosum is thinned and has WMHs in both genu and splenium (arrows in **B**). WMHs become confluent in the frontal and parietal regions in the postmortem T2 scan of the right hemisphere **(C)**. **(D–F)** Segmentation of the lateral ventricles and subcortical nuclei on T1 scans acquired in 2016 **(D)** and 2017 **(E)**. White matter lesions in the right temporal lobe are observed on both *in vivo* MRI scans (arrow) as well as the postmortem T2^*^ scan **(F)**. **(G,H)** The MCP sign (arrows) on T2 scans acquired in 2016 **(G)** and 2017 **(H)**. **(I,J)** Segmentation of the cerebellum on T1scans acquired in 2016 **(I)** and 2017 **(J)**.

Brain MRI during the initial evaluation showed a large middle cerebellar peduncles (MCP) sign, thin corpus callosum and white matter disease (WMD) in the splenium and genu of his corpus callosum and WMD in the right temporal lobe and periventricular areas (Figures 1A,D,G,I). Brain imaging at follow-up demonstrated severe brain atrophy with remarkable asymmetrical atrophy of the temporal lobes. Worsening of WMD in all areas including the insula and periventricular regions. The ventricles were larger and the corpus callosum was thinner when compared to the MRI taken 16 months prior (Figures 1B,E).

A quantitative analysis of brain atrophy was performed using volBrain software pipeline that implements a multi-atlas label fusion technique for segmenting the intracranial cavity, brain, lateral ventricle, cerebellar lobules, and subcortical nuclei (Manjon et al., 2014; Manjon and Coupe, 2016; Romero et al., 2017) (Figures 1D,E,I,J). Age- and sex-specific normative data are also available (Coupe et al., 2017). White matter hyperintensities (WMHs) were segmented automatically on fluid attenuated inversion recovery (FLAIR) scans using the LST toolbox from SPM12 (Schmidt et al., 2012), followed by manual editing using ITKsnap (Weis et al., 1993).

Compared with age- and sex-specific normative data (Coupe et al., 2017), percentages of intracranial volume (ICV) for whole brain, whole brain white matter, cerebrum, cerebral white matter, brainstem, thalamus, and amygdala were lower than the 95% lower limits while those of cerebrospinal fluid (CSF) and lateral ventricles were above the 95% upper limits for both visits. The ICV percentages of other regions, including the gray matter, cerebrum gray matter, cerebellum, cerebellar gray matter, and other nuclei under the investigation (Table 2) were within the 95% limits for both visits.

**Table 2:** Percentages of change and brain regional volumes compared with age- and sex-specific normative data.

**Brain regions**	**Visit 1 (Age 67.9)**						**Visit 2 (Age 69.2)**						**Whole brain % of change**
	**Volume (cm** ^**3**^ **)**	**% of ICV**	**95% lower limit**	**95% upper limit**	**Left hemi. volume (cm** ^**3**^ **)**	**Right hemi. volume (cm** ^**3**^ **)**	**Volume (cm** ^**3**^ **)**	**% of ICV**	**95% lower limit**	**95% upper limit**	**Left hemi. volume (cm** ^**3**^ **)**	**Right hemi. volume (cm** ^**3**^ **)**	
Whole brain	947	**63.4**	75.8	86.4	–	–	908	**61.3**	75.1	85.7	–	–	−4.1
Gray matter	663	44.4	40.0	51.3	–	–	635	42.9	39.9	51.2	–	–	−4.2
White matter	283	**19.0**	29.3	41.6	–	–	272	**18.4**	28.7	41.0	–	–	−3.9
Cerebrum	814	**54.5**	65.3	75.4	**416**	**397**	779	**52.5**	64.7	74.8	**399**	**379**	−4.3
Gray matter	544	36.4	33.6	43.2	276	268	523	35.3	33.5	43.0	266	257	−4.0
White matter	269	**18.0**	26.6	37.4	**140**	**129**	256	**17.3**	26.1	36.9	**134**	**122**	−5.0
Cerebellum	114	**7.6**	7.8	10.3	**56.2**	57.8	111	**7.5**	7.7	10.3	**54.0**	**56.5**	−3.1
Gray matter	102	6.8	5.6	8.2	50.4	51.8	98	6.6	5.6	8.2	48.2	49.9	−4.0
White matter	11.9	**0.79**	1.3	3.0	**5.8**	**6.1**	12.4	**0.84**	1.2	3.0	**5.9**	**6.6**	4.8
Brainstem	19.1	**1.3**	1.4	1.9	–	–	18.7	**1.3**	1.4	1.9	–	–	−2.4
CSF	546	**36.6**	13.6	24.2	–	–	574	**38.7**	14.3	24.9	–	–	5.1
Lateral ventricles	78	**5.2**	0.60	3.04	**37.8**	**40.1**	98	**6.6**	0.72	3.16	**47.4**	**51.1**	26.4
Caudate	5.49	0.37	0.36	0.55	2.81	**2.67**	5.39	0.36	0.36	0.55	2.80	**2.60**	−1.8
Putamen	7.16	0.48	0.42	0.64	3.50	3.66	7.09	0.48	0.42	0.63	3.50	3.59	−1.0
Thalamus	7.05	**0.47**	0.59	0.79	**3.47**	**3.57**	6.60	**0.45**	0.58	0.78	**3.22**	**3.38**	−6.3
Globus pallidus	2.30	0.15	0.13	0.20	1.19	1.11	2.05	0.14	0.13	0.20	1.08	0.97	−11.0
Hippocampus	6.96	0.47	0.44	0.63	3.50	3.46	6.48	0.44	0.44	0.62	3.25	3.23	−6.9
Amygdala	0.99	**0.07**	0.09	0.14	**0.53**	**0.46**	0.91	**0.06**	0.09	0.14	**0.48**	**0.44**	−7.6
WMHs	44	2.94	–	–	–	–	51	3.42	–	–	–	–	15.5

*Bold, outside of the 95% limit of the age- and sex-specific normative data*.

Over the 16 months between the two visits, the brain atrophied 4.12% while the lateral ventricles increased 26.4% and WMH volume increased 15.6%. Other regions atrophied substantially faster than the whole brain included the thalamus (−6.28%), globus pallidus (−10.95%), hippocampus (−6.95%), and amygdala (−7.58%). In contrast, the cerebellum (−2.40%), brainstem (−2.37%), caudate (−1.76%), and putamen (−1.03%) experienced slower than average atrophy rate (Table 2).

Post-mortem MRI showed confluent WMHs in the frontal and parietal lobes on T2 scan (Figure 1C) indicating further WMH expansion during the last 2 years of life and tissue loss in anterior temporal white matter (Figure 1F). Transverse relaxation rate, R2^*^, sensitive to iron content in the tissue, was estimated by fitting an ordinary lease squares function to the signal intensities of the multi-echo T2^*^ scan (Peran et al., 2007). Small focal increases in R2^*^ signals, consistent with cerebral microbleeds (CMBs), were observed in cerebral white matter as well as increased R2^*^ signals in the deep nuclei such as the putamen, globus pallidus, red nucleus, substantia nigra (SN), and cerebellar dentate nucleus.

### Genetic Evaluation

**Figure 2:**
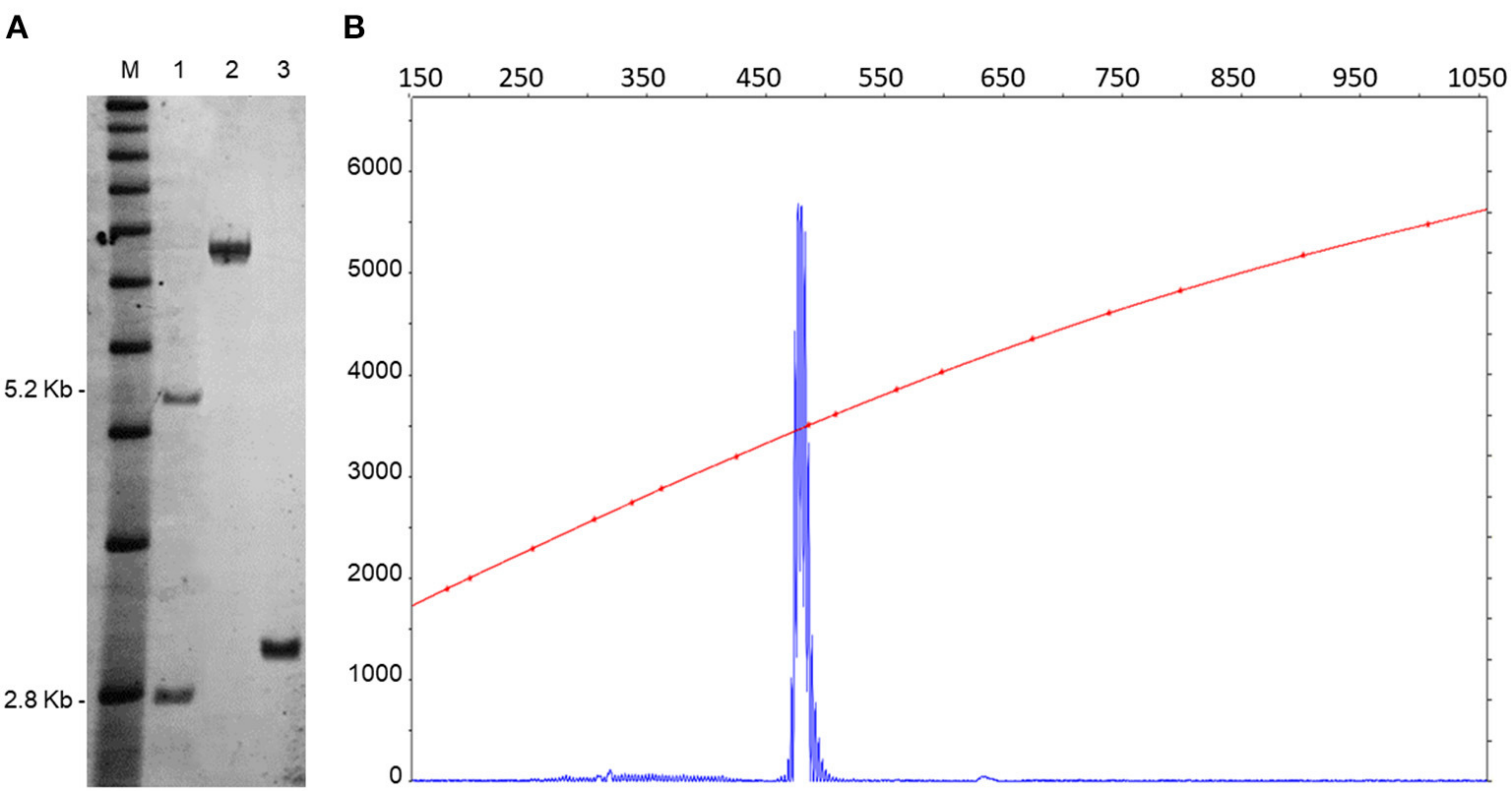
CGG repeat size and methylation status were determined by a combination of Southern blots **(A)** and capillary electrophoresis **(B)** on genomic DNA isolated from a female, negative control (lane 1), from a full mutation control (>200 CGG repeats) (lane 2). The SB analysis demonstrates the presence of an unmethylated premutation alleles in the proband (lane 3). M = DNA marker, 1 kb ladder. Normal unmethylated band (2.8 kb) and normal methylated band (5.2 kb) shown on the left. The electrophoregram **(B)** shows the presence of a single peak representing the premutation allele. The X-axis indicates the size of the allele in base pairs and the Y-axis indicates the fluorescence intensity of each allele.

DNA testing, including PCR and Southern blot analysis, as previously described (Filipovic-Sadic et al., 2010, Tassone et al., 2008), revealed the presence of a premutation allele of 85 CGG repeats. *FMR1* mRNA expression levels, measured by real time qRT-PCR (Tassone et al., 2000), were 2.5-fold higher than normal (2.58 StErr 0.04) (Figure 2). Genotyping for *APOE* allelic variants were carried out using TaqMan® assay genotype. An *APOE* ε3/ ε4 variant was documented.

### Postmortem Brain Assessment

**Figure 3:**
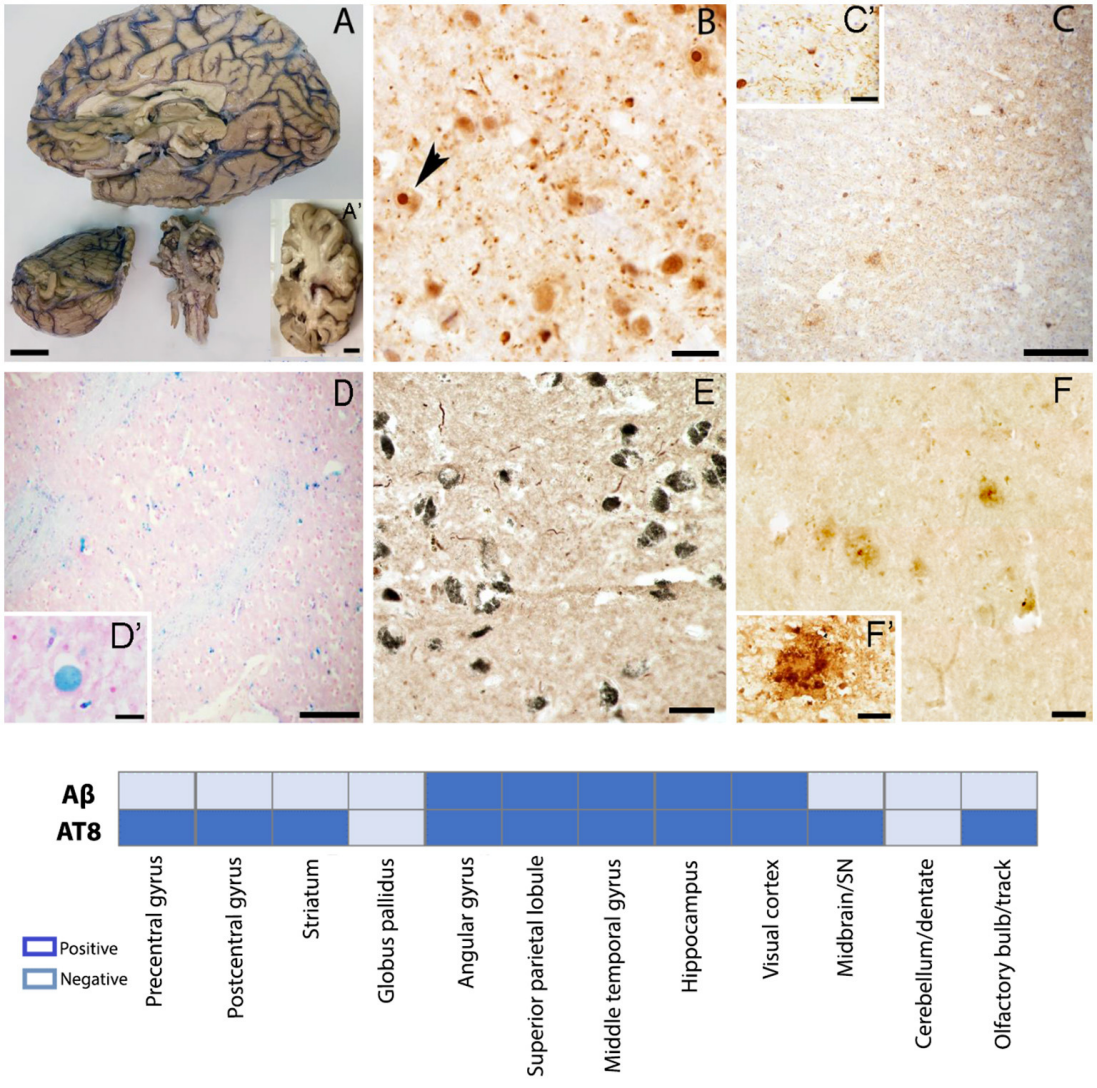
Gross and microscopic brain evaluation. **(A)** Severe frontal and temporal lobes atrophy, thin corpus callosum, sclerotic changes in basilar artery. **(B)** Ubiquitin positive intranuclear inclusions in the post central gyrus confirming definite diagnosis of FXTAS. **(C)** Tau positive plaques and neuropils in the prefrontal gyrus. **(D)** Iron deposits in the globus pallidus. **(E)** Tau positive neuropils in the SN **(F)** Amyloid β positive plaques in the middle temporal gyrus. **(A–A')** scale bar 2 cm; **(B)** scale bar 50 μm; **(C,D)** scale bar 200 μm; **(C')** scale bar 10 μm;; (**D** insert) scale bar 20 μm; **(E)** scale bar 50 μm; **(F)** scale bar 50 μm; **(F')** scale bar 20 μm, Graph shows a summary of brain regions sampled for histopathology evaluation. Aβ, amyloid beta; AT8, phosphorylated Tau.

The right hemisphere was fixed in 10% formalin for 8 weeks before dissection. After fixation gross descriptions were taken and the right hemisphere was coronally sectioned. Gross anatomy showed atrophy in the frontal and temporal lobes (Figure 3A), as well as mild ventricular enlargement, more predominant posteriorly. No evidence of major haemorrhagic strokes, infarctions, or abnormal growth were found grossly. The SN was well pigmented. Evidence of mild atherosclerosis was found within the basilar artery (Figure 3A).

Upon coronal sectioning, select anatomic areas were sampled from the frontal cortex, basal ganglia, inferior parietal cortex, temporal cortex, hippocampal formation including CA1, CA2 and entorhinal cortex, occipital cortex (Brodmann areas 17/18), hemisection of midbrain including SN and cerebellum, and then immersed in 30% sucrose in Tris-buffered saline (TBS). Once saturated, samples were embedded in optimal cutting temperature (OCT) compound (Fisher,USA), and frozen at −80°C. Sections were cut in cryostat at 14 μm thickness.

Our sampling deviated from the current recommendation for the evaluation of AD changes (Montine et al., 2012); since we did not include the middle frontal gyrus and the superior temporal gyrus in our analysis. In addition, we did not use the Consortium to Establish a Registry for AD (CERAD) (Mirra et al., 1991) protocol for neuritic plaque scoring. Hematoxylin and eosin stain was used for the assessment of WMD and CMBs, recently described as a common pathologic feature of FXTAS (Salcedo-Arellano et al., 2021).

Prior to immunostaining, slides were washed in TBS, hydrated in ethanol 100–50%, followed by antigen retrieval and incubation in 3% hydrogen peroxide, permeabilized and blocked in a TBS based solution (75% TBS, 15% Triton, 10% serum) for 2 h. Rabbit anti-ubiquitin (1:150; Dako, Glostrup, Denmark) was used to identify intranuclear inclusions commonly described in neurons and astrocytes, the major pathologic hallmark of FXTAS. A mouse monoclonal anti-phosphorylation clone AT8 tau [1:200; Invitrogen MN 1020 (Ser202, Thr205)] and a purified mouse anti-α-Synuclein, pY125 (1:200; BD Pharmingen) were used to evaluate tau aggregates and to confirm the presence of Lewy bodies in the SN respectively. A polyclonal rabbit anti-β amyloid 1–42 (ab10148, 1:100; Abcam, Cambridge, UK) was used to evaluate amyloid plaques and cerebral amyloid angiopathy (CAA), slides were incubated overnight at −4°C. Pearls Prussian blue (Meguro et al., 2007), was completed for the assessment of iron bound to hemosiderin, known to correspond to chronic microhaemorrhages and iron deposits.

We found ubiquitin-positive intranuclear inclusions in neurons and astrocytes of the prefrontal cortex (Figure 3B), confirming the definite diagnosis of FXTAS. We also noted either neurofibrillary tangles and/or neuropils and/or neuritic plaques in all the studied areas except for the globus pallidus and the cerebellum (Figure 3C). Using Braak scoring (Braak and Braak, 1995) for tau pathology a Braak stage V–VI suggests the co-occurrence of Alzheimer-type pathology. High amounts of iron deposits were visualized in the globus pallidus and the putamen consistent with MRI findings (Figure 3D). CMBs were noticed in the precentral gyrus, middle temporal gyrus, visual cortex, and brainstem. Tau aggregates were found in the neuromelanin containing neurons of the SN; however, no Lewy bodies were identified by α-synuclein immunostaining (Figure 3E), excluding a pathological diagnosis of concomitant Lewy body disease based on current criteria. Extracellular deposition of β-amyloid (core/diffuse plaques) was found in cortical areas and the hippocampus (Figure 3F), but absent in the basal ganglia, brainstem, and cerebellum. β-amyloid assessment met Thal phase (2) criteria (Thal et al., 2002); scattered β-amyloid positivity in leptomeningeal and cortical vessels, and capillaries, was also seen, but insufficient to fully assess CAA.

### Patient Perspective

Informed consent approved by the University of California Davis IRB committee was obtained at the time of clinical evaluation, for the use of neurocognitive and genetic testing for research purposes (IRB#254134). Brain donation was approved under IRB#215292. The final manuscript was provided for review and acceptance by the family for scientific publication.

## Discussion

Both, FXTAS and AD are neurodegenerative diseases that share overlapping cognitive deterioration that includes deficits in information processing speed, working memory and executive function (Grigsby, 2008) and both are known to have a slow progression (Hagerman and Hagerman, 2016, Vermunt et al., 2019) with preclinical phases. A prior postmortem brain study in females with the *FMR1* premutation reported concomitant FXTAS and AD (Tassone et al., 2012). In a small cohort of premutation carriers with and without FXTAS, at least one *APOE* ε*4* allele was found in 32% of those with FXTAS and it is hypothesized to serve as a genetic risk factor for developing FXTAS (Silva et al., 2013). However, the prevalence of coexistent FXTAS-AD pathology remains unknown. Now, we present a case of rapid motor and cognitive decline. By the time of his first evaluation in our clinic 5 years after his onset of memory and motor deficits, he met clinical criteria for probable Major Neurocognitive Disorder and used a mobility aid. His cognitive and motor abilities continued to decline steeply until death 4 years later.

Neuropsychiatric assessment plays a pivotal role in the early identification of cognitive impairment and neurological degeneration, especially in the prodromal and subsequent phases of AD (Zec, 1993). His neuropsychiatric tests showed reduced executive function abilities including a decrease across all WAIS-IV domains, especially working memory and verbal comprehension even at baseline. His MMSE score of 15, qualified for moderate dementia and further indicated significant impairment in cognitive function, which has been identified as a predictor of AD-associated mortality (Larson et al., 2004). Taken together, his symptoms supported the clinical diagnosis and expected progression of AD from mild to moderate memory issues within 1 year.

The patient's first scan presented with common findings of FXTAS including an MCP sign, thin corpus callosum and WMH (Jacquemont et al., 2003) but his cognitive deficits were strikingly severe for an initial FXTAS visit. A decrease in total brain and cortical volume, along with a significant decrease in temporal structure volume, all found to be related to atrophy in AD (Double et al., 1996), were identified in the scans of *in vivo* brain structures in addition to postmortem imaging and tissue examination. The increase of WMH and the ventricular size was also remarkable. Additionally, postmortem imaging revealed small focal increases in R2^*^ signals, consistent with CMBs in the cerebral white matter consistent with the pathological findings in multiple cortical regions and the SN. Postmortem MRI also revealed increased R2^*^ signals in the deep nuclei such as the putamen and the globus pallidus which presented with high amounts of hemosiderin positive deposits (Figures 1A–J). The brain atrophy in this case is faster than what is typically reported with FXTAS, and we hypothesize that those with FXTAS and coexistent Alzheimer-type pathology or perhaps the influence of the *APOE* ε4 alleles may contribute to a faster clinical decline compared to those with FXTAS alone. A prior report by Aydin et al., of a patient with FXTAS and faster than expected cognitive decline of the Alzheimer-type also demonstrated atrophy of the temporal lobes, however, the patient did not have an *APOE* ε4, FXTAS symptoms developed after age 70 and cognitive decline became apparent 10-years after the initial FXTAS diagnosis (Aydin et al., 2020). Both cases share a history of depression. Additional risk factors such as the use of anti-coagulants and vascular disease may have facilitated the progression of brain degeneration and FXTAS symptoms in this case. Such additional factors could have further exacerbated the progression of cognitive decline than just FXTAS and AD alone.

There are two remarkable histopathology findings to be highlighted in this case. First, the absence of Lewy bodies in the neuromelanin-containing neurons of the SN was unexpected since the patient presented mixed tremor in association with bradykinesia and masked facies (Figure 3E). However, FXTAS often presents with classic Parkinsonism in the absence of Parkinson's Disease (Salcedo-Arellano et al., 2020). Second, the contrasting burden between Aβ and tau proteins. While the studied brain regions had very few and in some sampled regions absence of amyloid deposits and scattered CAA, tau aggregates were found widely (Figures 3C,F) except for the globus pallidus and cerebellum (see graph in Figure 3 for additional information), a neuropathologic finding that has been observed in about 2–10% of cases in large autopsy studies and recognized as primary age-related tauopathy (PART) (Crary et al., 2014). We do not discard this alternative diagnosis in this case; however, the severe cognitive impairment in multiple domains and the inability of the patient to perform independent activities of daily living is more in accordance with our initial hypothesis of FXTAS-AD. Findings from the postmortem evaluation suggest the coexistence of FXTAS-AD, however, a definite pathological diagnosis will require further evaluation, including CERAD scoring, which was omitted due to differences in tissue thickness requirement.

## Data Availability Statement

The raw data supporting the conclusions of this article will be made available by the authors, without undue reservation.

## Ethics Statement

The studies involving human participants were reviewed and approved by Univeristy of California Davis IRB. The patients/participants provided their written informed consent to participate in this study. Written informed consent was obtained from the individual(s) for the publication of any potentially identifiable images or data included in this article.

## Author Contributions

MS-A: conception, organization, and execution of the research project (brain dissection, staining, microscopic analysis, and imaging). Writing of the first draft of the manuscript. DS: execution of the research project (tissue preparation, staining and imaging). Writing of the first draft of the manuscript. JW: execution of the research project (MRI analysis). Writing of the first draft of the manuscript. YM: execution of the research project (study coordinator, MRI acquisition, tissue preparation, staining, microscopic analysis, and imaging). Writing of the first draft of the manuscript. CC: writing of the first draft of the manuscript. PJ: execution of the research project (tissue preparation). Writing of the first draft of the manuscript. AS: execution of the research project (neuropsychiatric assessment). Writing of the first draft of the manuscript. FT: execution of the research project (genetic evaluation). Writing of the first draft of the manuscript. RH and VM-C: conception, organization, and execution of the research project. Review and critique of the manuscript. All authors contributed to the article and approved the submitted version.

## Funding

This research was supported by funds from the National Institute of Neurological Disorders and Stroke (NINDS) grant R01 1NS107131, the National Institute of Child Health and Human Development (NICHD) grant R01 HD036071, the MIND Institute Intellectual and Developmental Disabilities Research Center P50 HD103526, Shriners Hospitals for Children – Northern California, and the Victor E. LaFave III Memorial FXTAS Fund.

## Conflict of Interest

RJH has received funding from Zynerba, Ovid, and the Azrieli Foundation for carrying out treatment studies in patients with fragile X syndrome (FXS). She has also consulted with Fulcrum, Ovid, and Zynerba regarding treatment studies in individuals with FXS. FT has received funds from Asuragen and Zynerba for studies in FXS and associated disorders. VM-C has consulted with Paxmedica and received funding from Zynerba for organization of conferences in FXS and associated disorders. The remaining authors declare that the research was conducted in the absence of any commercial or financial relationships that could be construed as a potential conflict of interest.

## Publisher's Note

All claims expressed in this article are solely those of the authors and do not necessarily represent those of their affiliated organizations, or those of the publisher, the editors and the reviewers. Any product that may be evaluated in this article, or claim that may be made by its manufacturer, is not guaranteed or endorsed by the publisher.
